# Angiosarcoma of an Arteriovenous Fistula for Hemodialysis in a Kidney Transplant Recipient Affected by Lowe's Syndrome

**DOI:** 10.1155/2020/9734635

**Published:** 2020-04-13

**Authors:** V. D'Ambrosio, P. Silvestri, F. Aureli, A. Sturniolo, G. Grandaliano, P. M. Ferraro

**Affiliations:** ^1^Università Cattolica del Sacro Cuore, Rome, Italy; ^2^U.O.C. Nefrologia, Fondazione Policlinico Universitario A. Gemelli IRCCS, Rome, Italy

## Abstract

*Objective/Background*. To describe an uncommon, life-threatening condition such as angiosarcoma of a fistula for hemodialysis occurring in a transplant recipient affected by Lowe's syndrome. *Summary*. We present the case of a 56-year-old male kidney transplant recipient affected by Lowe's syndrome, also known as oculocerebrorenal syndrome, a rare X-linked disorder characterized by congenital cataracts, hypotonia, intellectual disability, and Fanconi-like renal tubular dysfunction, who was diagnosed with angiosarcoma of a functioning arteriovenous fistula for hemodialysis. *Conclusion*. Angiosarcoma is a rare soft tissue tumor, and only 22 cases of angiosarcoma of arteriovenous fistulae were described so far; although a correlation between Lowe's syndrome and a higher risk of tumor compared to the general population has not been described so far, the mechanisms of disease causation could be an interesting starting point for future studies on a possible connection between the two events.

## 1. Introduction

Angiosarcomas (AS) arising from arteriovenous fistulas (AVFs) for hemodialysis are rare events. Oskrochi et al. in a recent review described 22 cases, 19 of which were post-transplant recipients and 18 were on immunosuppression therapy [1]. Multiple possible pathogenic mechanisms, such as immunosuppressive therapy and altered lymphatic flow, are already described in the literature, although a certain etiology has not been established yet.

We present the case of a 56-year-old male kidney transplant recipient affected by Lowe's syndrome, also known as oculocerebrorenal syndrome, a rare X-linked disorder characterized by congenital cataracts, hypotonia, intellectual disability, and Fanconi-like renal tubular dysfunction, who was diagnosed with angiosarcoma of a functioning arteriovenous fistula for hemodialysis. There is no evidence of an increased risk of tumor in these patients except for subcutaneous benign fibromas; however, the disease's molecular pathogenesis could potentially play a role.

## 2. Case Report

A 56-year-old male kidney transplant recipient affected by Lowe's syndrome was admitted to our hospital for a hematoma on his left forearm.

The patient had a history of end-stage renal disease (ESRD) and had undergone hemodialysis for 11 years through a distal AVF on his left forearm. In 2013, the patient received a deceased-donor kidney transplant and has been treated with immunosuppressive therapy with tacrolimus 1 mg + 0.5 mg daily ever since. He also had a recent history of ischemic stroke and accidental fall at home which caused a trauma of his left wrist.

A week before the admission, the patient was evaluated by the surgical team of our department because of a painful and swelling mass on his left forearm where the AVF for hemodialysis was originally created.

The enlarging mass first appeared two months before and was treated in another hospital as a complicated AVF, and thus the vascular access was closed.

During the hospitalization in our Nephrology Department, the patient underwent surgical revision of the hematoma and ligation of the distal radial artery. A left wrist radiogram was also performed which showed an area of diaphyseal osteolysis of the radius surrounded by a voluminous soft-tissue swelling. After a few days, the radial artery required ligation right after its origin from the brachial artery because of enlarging hematoma.

After a short time of apparent stability, the patient was admitted once again to our emergency department because of active bleeding from the surgical site which required urgent evacuation of the hematoma. Although the specific source of the hematoma was undetectable, the hemostasis was achieved using surgical glue devices. A surgical reduction of the brachial artery's diameter was also necessary. The hospitalization was complicated by two events of severe anemia which required blood transfusions.

During the follow-up, because of chronic anemia and worsening of the forearm lesion, the patient was readmitted to our department. A Doppler US was performed; however, detailed characterization of the mass required further imaging with computed tomography (CT). Because of high suspicion of malignancy (spread vascular enhancement and pathologic fracture of the radius), a bone biopsy of the fractured radius was required, which then confirmed the presence of epithelioid sarcoma. A total-body CT scan was performed with evidence of enlarged ipsilateral axillary lymph nodes.

After a consultation with the oncologist and the radiotherapist, considering the extension of the lesion, comorbidities, and scarce compliance of the patient, chemo and radiotherapy were ruled out and a surgical approach was favored. The patient was then submitted for left arm amputation (above elbow). The hospitalization was complicated by *Clostridium difficile* infection treated with oral vancomycin and *Proteus mirabilis* urinary tract infection treated with intravenous meropenem. Four months following amputation, the patient was found to have metastatic disease to both lungs, the left iliac wing, and the duodenum. Five months later, the patient died.

## 3. Discussion

Angiosarcoma is a rare yet very aggressive tumor that arises from endothelial tissue. AS arising from AVFs are even rarer events. Steal syndrome, aneurysms, ischemia, and thrombosis are well-known blood-flow related complications of AVFs. Malignancies such as angiosarcomas may occur in both functioning and nonfunctioning fistulae, and although they can occur spontaneously, multiple risk factors have been identified. Chronic lymphedema may play an important role; as a matter of fact, when creating an AVF, not only the blood flow is altered but the lymphoid dynamics of soft tissues surrounding the AVF itself changes as well. This mechanism leads to the creation of a localized immunosuppressed environment which contributes to AS occurrence [2, 3]. AVF creation and altered blood flow also lead to hypoxia and wall shear stress, both strong proinflammatory stimuli. Hypoxic and ischemic injury promote inflammation, angiogenesis, and proliferation by stimulating the release of cytokines such as hypoxia-inducible factor-1 alpha (HIF-1*α*), immediate-early responsive gene (IEX-1), vascular endothelial growth factor (VEGF), interleukin-1 beta (IL-1*β*), tumor necrosis factor alpha (TNF*α*), and interferon gamma (IF*γ*) from endothelial cells. These cytokines function both as proliferation stimuli and leukocyte attractant, ultimately resulting in intima hyperplasia and blood flow-related complications of AVFs, but could also potentially play a role in AS pathogenesis [4]. In transplant recipients or patients undergoing immunosuppressive therapy for other reasons, an important risk factor for malignancy occurrence is immunosuppression; it is well known that immunosuppressed populations have a double incidence rate of all malignancies compared to the normal population [5, 6].

Our patient had many risk factors that could have been potentially implicated in the pathogenesis of AS of the fistula, including being affected by a rare disease whose molecular pathogenesis interferes with cellular migration and differentiation of many cell types.

Lowe's syndrome (oculocerebrorenal syndrome) is a rare X-linked disorder characterized by involvement of the eyes, central nervous system, and kidneys.

Dense congenital cataract is the most common and earliest eye manifestation; nystagmus, microphthalmos, enophthalmos, severe glaucoma, strabismus, retinal dystrophy, secondary corneal scarring, and calcific band keratopathy with keloid formation may be present and worsen visual damage, ultimately progressing to blindness.

In almost all affected males, generalized hypotonia is present soon after birth (absent deep tendon reflexes, poor head control, sucking and swallowing problems leading to feeding difficulties, and delayed growth), but it may improve with age. Seizures and behavior problems are frequent, as much as intellectual impairment.

Renal pathology is characterized by proximal renal tubular dysfunction resulting in an abnormal loss of bicarbonate, sodium, potassium, amino acids, organic acids, albumin, calcium, and L-carnitine in the urine.

Low-molecular-weight proteinuria (LMW proteinuria) and albuminuria are present as first clinical manifestations, followed by polyuria and renal tubular acidosis. Some patients may develop full-spectrum renal Fanconi syndrome (bicarbonaturia and renal tubular acidosis, phosphaturia with hypophosphatemia and renal rickets, sodiuria and potassiuria, glycosuria, polyuria, and apparent urine-concentrating defect), and others may develop LMW proteinuria, hypercalciuria with nephrocalcinosis, and nephrolithiasis similar to Dent's disease. Chronic tubular injury eventually leads to glomerulosclerosis and thus to a decline in glomerular filtration rate (GFR). End-stage renal disease (ESRD) usually occurs between the second and the fourth decade of life.

Other clinical manifestations may be short stature, feeding difficulties and gastrointestinal problems such as gastroesophageal reflux and chronic constipation, scoliosis, arthritis, tenosynovitis and subcutaneous benign fibromas, cryptorchidism, and dental malformations. Superficial cysts are also frequent, especially on the skin and in the mouth, but they can also occur in the kidneys and brain. Lowe patients may also have an intrinsic platelet defect leading to prolonged bleeding time [7].

Almost all of the disease's manifestations may be explained by its molecular pathogenesis. Lowe's syndrome is caused by a hemizygous pathogenic variant in OCRL, a gene located on the X chromosome (locus q26.1) decoding for inositol polyphosphate 5-phosphatase or phosphoinositide 5-phosphatase OCRL-1/INPP5F. The same gene is also involved in type 2 Dent's disease, a disease characterized by LMW proteinuria, hypercalciuria, nephrolithiasis, nephrocalcinosis, and progressive renal failure [8].

There are 10 different isozymes of phosphoinositide 5-phosphatase (PI 5-phosphatase/INPP5P) [9], and each protein is expressed in specific cell types and interacts with specific pathways by regulating intracellular concentrations of their substrates. All PI 5-phosphatase, except for INPP5A, catalyze the dephosphorylation of two lipids: PI-(3,4,5)-trisphosphate (PI-(3,4,5)-P3) and PI-(4,5)-bisphosphate (PI-(4,5)-P2) [10, 11]. They therefore act as potential tumor suppressors in tumor cells by reducing the intracellular concentrations of PI-(3,4,5)-P3 [12] and by increasing intracellular concentrations of PI-(3,4)-P2.

Reduced or absent activity of PI 5-phosphatases leads to intracellular accumulation of their substrate. In Lowe's syndrome, OCRL-1/INPP5F codes for a very specific phosphatase and its altered function lead to phosphatidylinositol (4,5) bisphosphate [PtdIns (4,5) P2] increase [13], a molecule that has many signaling functions in endocytosis, exocytosis, migration, and adhesion by interacting with cellular compounds such as scaffold proteins, ion channels, and cytoskeleton proteins [14]. PtdIns (4,5) P2 thus interferes with many cellular processes such as differentiation and cell migration by altering cell membrane composition, cytoskeletal organization, and ciliary function, which could partially explain the ophthalmologic complications. Recent evidence demonstrates that PtdIns (4,5) P2 is involved in endocytosis and lysosomal-autophagic pathway and its accumulation may alter intracellular protein trafficking and tubular transport. This mechanism could explain LMW proteinuria and tubular defects [10].

Although the tumor promotor versus tumor suppressor role of 5-phosphatases has been discussed in the literature, correlating some of them to cancer (e.g., overexpression of SYNJ2 is correlated with breast cancer growth and metastases) [15], there is no association between OCRL-1/INPP5F and cancer [16].

However, in Lowe's syndrome, the intracellular alterations cited above are present in many different cell types, from kidney, brain, lung, ovary, and testis cells to fibroblasts which share their mesenchymal origin with endothelial cells.

## 4. Conclusion

Among the many risk factors that contributed to the development of AS from our patient's fistula, Lowe's syndrome could potentially play a role, although a direct link between the two events has not been described yet.

Because of the poor prognosis of AS, an early diagnosis is crucial to a successful treatment or at least to a prolonged survival. When bleeding or painful masses arise from the site of AVFs in transplant recipients, angiosarcomas must be ruled out among the differential diagnoses, especially in patients with other comorbidities.

## References

[1] Oskrochi Y., Razi K., Stebbing J., Crane J. (2016). Angiosarcoma and dialysis-related arteriovenous fistulae: a comprehensive review. *European Journal of Vascular and Endovascular Surgery*.

[2] Bordea C., Cortina-Borja M., Wojnarowska F., Morris P. J. (2001). Distribution of upper limb skin cancers in relation to arteriovenous fistula side in renal transplant recipients. *Transplantation*.

[3] Conlon P. J., Daly T., Doyle G., Carmody M. (1993). Angiosarcoma at the site of a ligated arteriovenous fistula in a renal transplant recipient. *Nephrology Dialysis Transplantation*.

[4] Fitts M. K., Pike D. B., Anderson K., Shiu Y.-T. (2014). Hemodynamic shear stress and endothelial dysfunction in hemodialysis access. *The Open Urology & Nephrology Journal*.

[5] Engels E. A., Pfeiffer R. M., Fraumeni J. F. (2011). Spectrum of cancer risk among US solid organ transplant recipients. *JAMA*.

[6] Grulich A. E., van Leeuwen M. T., Falster M. O., Vajdic C. M. (2007). Incidence of cancers in people with HIV/AIDS compared with immunosuppressed transplant recipients: a meta-analysis. *The Lancet*.

[7] Lewis R. A., Nussbaum R. L., Brewer E. D., Adam M. P., Ardinger H. H., Pagon R. A. (1993). Lowe syndrome. *GeneReviews®*.

[8] Devuyst O., Thakker R. V. (2010). Dent’s disease. *Orphanet Journal of Rare Diseases*.

[9] Blero D., Payrastre B., Schurmans S., Erneux C. (2007). Phosphoinositide phosphatases in a network of signalling reactions. *Pflügers Archiv—European Journal of Physiology*.

[10] Erneux C., Edimo W. s. E., Deneubourg L., Pirson I. (2011). SHIP2 multiple functions: a balance between a negative control of PtdIns(3,4,5)P3level, a positive control of PtdIns(3,4)P2production, and intrinsic docking properties. *Journal of Cellular Biochemistry*.

[11] Erneux C., Ghosh S., Raquel Ramos A., Elong Edimo W. s. (2016). New functions of the inositol polyphosphate 5-phosphatases in cancer. *Current Pharmaceutical Design*.

[12] Toker A., Rameh L. (2015). PIPPing on AKT1: how many phosphatases does it take to turn off PI3K?. *Cancer Cell*.

[13] Zhang X., Hartz P. A., Philip E., Racusen L. C., Majerus P. W. (1998). Cell lines from kidney proximal tubules of a patient with Lowe syndrome lack OCRL inositol polyphosphate 5-phosphatase and accumulate phosphatidylinositol 4,5-bisphosphate. *Journal of Biological Chemistry*.

[14] Ramos A. R., Elong Edimo W., Erneux C. (2018). Phosphoinositide 5-phosphatase activities control cell motility in glioblastoma: two phosphoinositides PI(4,5)P2 and PI(3,4)P2 are involved. *Advances in Biological Regulation*.

[15] Ben-Chetrit N., Chetrit D., Russell R. (2015). Synaptojanin 2 is a druggable mediator of metastasis and the gene is overexpressed and amplified in breast cancer. *Science Signaling*.

[16] Rudge S. A., Wakelam M. J. O. (2016). Phosphatidylinositolphosphate phosphatase activities and cancer. *Journal of Lipid Research*.

